# Neighbourhood Ethnic Density Effects on Behavioural and Cognitive Problems Among Young Racial/Ethnic Minority Children in the US and England: A Cross-National Comparison

**DOI:** 10.1007/s11113-017-9445-1

**Published:** 2017-09-05

**Authors:** Nan Zhang, Jennifer L. Beauregard, Michael R. Kramer, Laia Bécares

**Affiliations:** 1 Cathie Marsh Institute for Social Research (CMI), School of Social Sciences, The University of Manchester, Manchester, M13 9PL UK; 2 Rollins School of Public Health, Emory University, Claudia Nance Rollins Building 1518 Clifton Road, NE, Atlanta, GA 30322 USA; 3 Department of Social Statistics, School of Social Sciences, The University of Manchester, Manchester, M13 9PL UK

**Keywords:** Ethnic density, Child development, Neighbourhood effects, England, United States

## Abstract

Studies on adult racial/ethnic minority populations show that the increased concentration of racial/ethnic minorities in a neighbourhood—a so-called ethnic density effect—is associated with improved health of racial/ethnic minority residents when adjusting for area deprivation. However, this literature has focused mainly on adult populations, individual racial/ethnic groups, and single countries, with no studies focusing on children of different racial/ethnic groups or comparing across nations. This study aims to compare neighbourhood ethnic density effects on young children’s cognitive and behavioural outcomes in the US and in England. We used data from two nationally representative birth cohort studies, the US Early Childhood Longitudinal Study-Birth Cohort and the UK Millennium Cohort Study, to estimate the association between own ethnic density and behavioural and cognitive development at 5 years of age. Findings show substantial heterogeneity in ethnic density effects on child outcomes within and between the two countries, suggesting that ethnic density effects may reflect the wider social and economic context. We argue that researchers should take area deprivation into account when estimating ethnic density effects and when developing policy initiatives targeted at strengthening and improving the health and development of racial and ethnic minority children.

## Introduction

It is well documented that most ethnic minority groups in the United States (US) and England fare less well across a wide range of health outcomes than their majority White peers (e.g. Jackson and Mare 2007; Smith et al. 2000; Williams and Collins 2001). Although most of the literature on racial/ethnic health inequalities focuses on adults, a large body of research has also documented marked racial/ethnic inequalities in the patterning of early childhood health and developmental outcomes such as birthweight (Kelly et al. 2009; Teitler et al. 2007), breastfeeding (Kelly et al. 2006b), developmental milestones (Kelly et al. 2006a), socioemotional difficulties (Zilanawala et al. 2015b), obesity (Zilanawala et al. 2015a), cognitive scores (Panico and Kelly 2007), and asthma (Nelson et al. 1997; Panico et al. 2007; Weitzman et al. 1990). For both children and adult populations, racial/ethnic inequalities in health are largely explained by reduced socioeconomic status of racial/ethnic minority groups (Nazroo 2000; Williams 1999; Zhang and Wang 2004), including at the area-level (Iceland et al. 2011; Karlsen et al. 2002; Omi and Winant 2014).

However, the impact of individual socioeconomic status may be moderated by the wider socioeconomic context. Studies have shown a protective effect for members of racial/ethnic minority groups of living in neighbourhoods with high concentrations of other ethnic minority residents after adjusting for area deprivation, a phenomenon called the ethnic density effect. Studies on the ethnic density effect have centred on adult populations in single nations, with only one study to date comparing across countries (Bécares et al. 2012a). In the present study, we use a cross-national perspective to examine whether ethnic density effects are also present for children living in two contrasting but comparable national settings: the US and England.

### Ethnic Density Effects on Health

Neighbourhoods where people reside are an important determinant of health and health inequalities (Boyle and Lipman 2002; Pickett and Pearl 2001). A common feature of modern residential environments is the spatial concentration of families by race, ethnicity, immigration status, and class. Racial/ethnic minorities in both the US and England are more likely than Whites to live in the most deprived neighbourhoods in terms of economic and physical resources (Iceland et al. 2011; Karlsen et al. 2002; Omi and Winant 2014). Particularly in the US, the concentration of racial/ethnic minority people in specific neighbourhoods is generally thought about in relation to segregation, highlighting the deleterious health effects and reduced opportunities of spatial stratification (Williams and Collins 2001). However, it is the concentration of poverty and not racial/ethnic minority concentration that is associated with detrimental health and social outcomes (Bécares et al. 2014). A growing body of literature has been examining the manner in which the clustering of ethnic minority people of the same group in a neighbourhood, a phenomena called the ethnic density effect, provides protective effects on health when adjusting for area-based material wealth or deprivation (Bécares et al. 2009, 2012b; Pickett and Wilkinson 2008; Shaw et al. 2012).

Although several pathways have been proposed, there are two main mechanisms by which ethnic density is hypothesised to protect the health of ethnic minority residents: (1) via increased social cohesion, and (2) through reduced exposure to racial discrimination, and a decreased strength in the negative association between experiences of racial discrimination and poor health. The first mechanism proposes that ethnic density generates higher neighbourhood cohesion through a stronger sense of community and belongingness (Bhugra and Becker 2005; Daley 1998; Halpern and Nazroo 2000; Smaje 1995). Ethnic group membership is often a basis for networks of social relations (Bankston and Zhou 2002), and a source of economic and moral support for second generations (Portes and Zhou 1993). Existent research has already documented the association between increased ethnic density and higher community social cohesion (Bécares et al. 2011), and between higher community social cohesion and lower morbidity (Berkman and Kawachi 2000; Fone et al. 2007; Stafford et al. 2003).

The second mechanism postulates that ethnic density is associated with health through a decrease in the prevalence of experienced interpersonal racism. Experiences of racism have been widely documented to have a detrimental impact on health (Harris et al. 2006; Karlsen and Nazroo 2002; Krieger and Sidney 1996; Paradies 2006), and studies on the associations between ethnic density, racism, and health among adults have provided support for this mechanism (Bécares et al. 2009, 2012a). For older children, Astell-Burt et al. (2012) found a null association between own ethnic density and poor psychological well-being among adolescents aged 11–16 from London after adjustment for neighbourhood deprivation. They suggested that racism, but not ethnic density and area deprivation, played an important role in adolescents’ psychological well-being. Hurd et al. (2013) have reported that increased ethnic density among African American adolescents was related to decreased internalising symptoms. But in a Dutch study among adolescents aged 11–18 years, Gieling et al. (2010) found a null association between ethnic density (measured as the proportion of pupils in class with ethnic minority status) and internalising problem behaviour.

Given the frequent association between the population density of ethnic minorities and lower areal socioeconomic status, the hypothesised health-protective effects of ethnic density must be considered after conditioning on individual and area-based material deprivation. Studies examining the individual cases of the US, the UK, and New Zealand report that ethnic density effects are negative and detrimental prior to adjusting for area-level deprivation, but the direction of the effect changes with such adjustment and ethnic density effects become protective (Bécares et al. 2011, 2013, 2014).

However, findings are not always consistent. In the case of African Americans in the US, studies show that, given the history of slavery and the distinctive residential segregation experienced by this group, in the highest levels of ethnic density the concentration of poverty, chronic underinvestment, and social isolation overpower any potential benefits that may emerge from ethnic density (Bécares et al. 2012b). Although racial/ethnic minority groups in England have not experienced the same levels of historic residential concentration as African Americans in the US, the influence of area-level deprivation in obscuring possible protective ethnic density effects is of great importance, and the adequate modelling of area-level deprivation is key in order to detect any ethnic density effects on health (Bécares et al. 2012a, b).

The US and England are two interesting contexts to compare in cross-national studies because they share stark inequalities in the health and socioeconomic characteristics of racial/ethnic minority populations when compared to their majority White populations. Despite these structural similarities in the existence of racial/ethnic inequalities, the particular composition of individual racial/ethnic minority groups in both countries is very different, with different countries of origin with varying patterns of colonisation, different historical periods and reasons for migration, and different patterns of internal forced migration. Thus, a comparative study can potentially uncover variation in the effect of membership in a given minority group when the surrounding historical and societal circumstances differ.

### Ethnic Minority Groups in England

In England, five ethnic minority groups are large enough to be studied in population representative health and social surveys: Black Caribbeans, Black Africans, Indians, Pakistanis, and Bangladeshis. Most Black Caribbean migration occurred in the post Second World War era due to labour shortages in England. The Black Caribbean population concentrated in urban areas, and the majority are currently located in four main metropolitan clusters: Greater London, which alone accounts for over half of the Black Caribbean population, Birmingham, Greater Manchester, and West Yorkshire (Peach 1998). Although migrants arrived to fill semi-skilled and unskilled employment gaps, Black Caribbean people have experienced occupational mobility since the 1950s, with significant numbers of their population working in a managerial or professional occupations (Connolly and White 2006).

The Black African presence in England is long-standing, rooted in the settlements established by Nigerian and Somali ex-seamen in ports such as London, Liverpool, Cardiff, and South Shields, starting in the late nineteenth century. These initial settlements were subsequently bolstered by the arrival of well-educated young migrants from Nigeria, Sierra Leone, and Ghana who came for educational purposes. The latest wave of Black African migration consisted of refugees seeking asylum, and started with the political instability of the 1970s and 1980s from countries such as Eritrea, Uganda, Somalia, Ethiopia, Angola, Congo, and Nigeria (Daley 1998). These different reasons for migration are reflected in the observed settlement patterns. For example, migrants who came for educational purposes and thus achieved a high socioeconomic status reside in middle-class neighbourhoods. In contrast, recent migration characterised by political asylum is reflected through patterns of concentration in highly segregated and deprived neighbourhoods (Daley 1998). As a group, Black Africans are disproportionately concentrated in social housing, with high levels of overcrowding, and with similar settlement patterns as those of Black Caribbean people (Daley 1998).

Indian migration to England has occurred across multiple waves. The initial phase occurred in the late 1950s and early 1960s with the arrival of Sikhs and Hindus from the Punjab region and the Gujarat area. In 1970, a second wave of Indian migrants from Uganda, Kenya, and Tanzania came to England, subsequent to their first migration from India to East Africa. The current Indian population has come for professional and white-collar employment, with large proportions of men in the top professional class (Peach 1998). A considerable proportion of Indian women are in the labour force, which increases the overall socioeconomic standing of Indian households.

Migration from Pakistan to England started in the early 1960s with a wave of unskilled migrants who came to fill textile jobs. The influx of Pakistani migrants increased immediately after the introduction of the 1962 Immigration Act, and decreased when voucher issuing was stopped in 1965 (Amin 2002). During the 1970s and 1980s, a wave of wives and children came to England to join their family members (Amin 2002). The majority of Pakistani people are predominantly in manual and blue-collar employment, and Pakistani women are less likely to participate in the labour force than Indian women. Thus, Pakistani households are on average of lower socioeconomic standing than Indian households.

The main wave of Bangladeshi migration to England started in the 1960s, and peaked after 1971 following the partition of Greater Pakistan, which turned the Province of Old East Bengal into Bangladesh. The initial wave consisted of male economic migrants, and increased thereafter with the arrival of their wives and dependants as occurred for the other South Asian groups (Peach 1998). Bangladeshi migrants first concentrated in inner London. Bangladeshi migrants brought their families to England later than did other South Asian groups, which has translated into the present youthful age structure of the Bangladeshi population. Currently, Bangladeshi people are found mainly in manual, blue-collar employment, and have settled in east London and Birmingham, areas characterised by high degrees of residential concentration and overcrowding (Peach 1998).

### Racial/ethnic Minority Groups in the US

In the US, the four racial/ethnic minority groups with sufficient numbers to be included in this study (Black or African American, Hispanic, Asian, and American Indian) made up 37.8% of the total population in 2014, and 48% of children under 18 (Colby and Ortman 2015). African-origin populations in the eighteenth and nineteenth centuries largely arrived in the US as a result of forced migration through the African slave trade, and the majority of Black Americans today can trace ancestry back to this legacy (Pollard and O’Hare 1999). More recently, migration of Black African people into the US followed new immigration policies over the 1960s to 1990s which enhanced US openness, allowed for more asylum-seeking refugees from conflict areas, and allowed for increased migration from underrepresented nations (Anderson 2015). The percent of Black Americans who are foreign-born almost tripled from 3.1 to 8.7% between 1980 and 2015, mostly due to increased migration from Caribbean and African nations with the largest numbers coming from Jamaica, Haiti, and Nigeria (Anderson 2015).

The label Hispanic refers to ethnicity and Hispanics may be of any race, although important heterogeneity exists among Hispanics by race as well as by country of origin. A portion of the contemporary American Hispanic population is descended from peoples who lived in what is now the Southwestern US well before the region was organised into states. The first wave of Hispanic migration to recognised US territories occurred during the California Gold Rush after the US–Mexico border was established at the end of the US–Mexican War in 1848. Thousands of Mexican migrants arrived in the US each decade for much of the remainder of the nineteenth century, and migration sharply increased starting around 1890 with acceleration of economic development in the Western US (Gutiérrez 2016). Over the course of the twentieth century, migration of Hispanic groups to the US has occurred mostly from Spanish-speaking countries in Latin America (Pollard and O’Hare 1999). Migration has accelerated since the 1960s due to political turmoil in Cuba and Central America, changes to US immigration policy in 1965, and economic opportunities for Hispanic migrants compared with those available in their home countries (Gutiérrez 2016).

Nearly two-thirds of US Hispanics in 2010 were of Mexican-origin, but there were also more than 1 million people each of Cuban, Salvadoran, Dominican, or Guatemalan origin (Motel and Patten 2012). The Hispanic ethnicity group also includes US citizens who move from Puerto Rico to the mainland. Some Hispanic groups cluster within specific areas of the US by country of origin, such as Cubans in Florida and Puerto Ricans in New York City (Motel and Patten 2012).

While Asian migration to the US began in the eighteenth century, most of this group immigrated relatively recently (Pollard and O’Hare 1999). Similar to other groups, Asian migration increased dramatically after the 1965 Immigration and Nationality Act; the number of Asians and Asian-Americans living in the US increased from roughly 500,000 in 1960 to 12.8 million in 2014. Among Asians in the US, the largest country-of-(ancestral)-origin groups are from India, China, the Philippines, Vietnam, and Korea. Reasons for migration include economic and educational opportunities, family reunification, and humanitarian protection (Zong and Batalova 2016).

American Indians and Alaskan Natives are descended from indigenous peoples who lived in North America prior to the arrival of Europeans. After European arrival in North America, indigenous populations declined dramatically due to disease and warfare. By 1890, the population stood at fewer than 250,000. American Indians have been subjected to forced migration out of their homelands and onto tribal lands as well as marginalisation through the withholding of citizenship rights until 1924 (Pollard and O’Hare 1999). Today, this group includes over 5 million American Indians or Alaska Natives with most residing in the western or southern US (Norris et al. 2012).

### Contrasting Socio-historical Context of Racial/Ethnic Groups

The histories of voluntary migration, forced migration, settlement patterns, and length of time in destination countries vary greatly between and within racial/ethnic groups in the US and England (Peach 1999). These distinct historical trajectories may further inform or contextualise contemporary exploration of health effects of ethnically dense neighbourhoods. Comparing and contrasting within as well as across national settings, there are racial/ethnic groups that experience high levels of spatial stratification and isolation such as African Americans and American Indians in the US; groups in more preliminary stages of spatial assimilation such as Asians in the US, and Pakistani and Bangladeshi groups in England; and groups that have increasingly assimilated over time such as Hispanics in the US and the Black Caribbean group in England. Groups can experience changes over time in divergent directions. In the US, Asians increasingly reside in less concentrated and less deprived neighbourhoods. In England, Pakistani and Bangladeshi children are more likely to continue to live in concentrated, deprived areas, whereas Indian and Black Caribbean children are experiencing transitions into less concentrated and deprived neighbourhoods.

We propose that these differences in residential settings, both within and across national contexts, will contribute not only to the patterns of health and development among children, but in the association between ethnic density, area deprivation, and health outcomes. Studies among adult populations show that ethnic density is often most beneficial for the most disadvantaged group and least protective for the health of the most advantaged group (Bécares 2014). We hypothesise that these patterns will hold for children as well, that the most disadvantaged groups receive the greatest benefit from the protective buffering properties of ethnic density effects since they experience the greatest societal disadvantages (lowest socioeconomic resources, high levels of racial discrimination) (Bécares et al. 2009). Differences in ethnic density effects reflect actual differences in the lived realities across racial/ethnic minority populations.

### Neighbourhood Effects and Child Health and Development

The examination of neighbourhood effects on health among the multiple racial/ethnic groups across national boundaries offers the opportunity to characterise common effects of residence with co-ethnics and to describe variations between and within nations. By contrasting and comparing ethnic density effects within and across countries, this study aims to shed light on the hypothesis that the most oppressed and disadvantaged racial/ethnic groups receive the greatest protection from the properties of ethnic density. Previous studies on the effect of ethnic density have predominantly focused on adult health and morbidity (e.g. Bécares et al. 2009, 2012a, b; Pickett and Wilkinson 2008) and on birth outcomes (e.g. Mason et al. 2011; McLafferty et al. 2012; Pickett et al. 2009; Shaw et al. 2010). Although a few studies have examined the effects of ethnic density on child outcomes, the association between ethnic density and young children’s health and development remains largely unexplored (Astell-Burt et al. 2012; Georgiades et al. 2007; Gieling et al. 2010; Hurd et al. 2013; Jensen and Rasmussen 2011; Leventhal and Shuey 2014). It is not obvious that associations observed in adulthood will be mirrored in childhood, and it is important to understand whether, and how, any salubrious or detrimental effects of residential environments on health vary across different life course stages. To the best of our knowledge, no study to date has examined ethnic density effects on child health and development through cross-national comparison.

This gap in the literature extends to a lack of information regarding the mechanisms that may explain these associations. With regard to racial/ethnic minority children, it is important to acknowledge the role of social stratification variables, such as social class and race/ethnicity, in shaping the neighbourhood contexts where child development occurs (Coll et al. 1996), both in terms of contextual and compositional characteristics. Neighbourhood effects on young children can operate across a myriad of mechanisms, including directly via neighbourhood resources (public and private services available in the neighbourhood, for example, parks, libraries, health care and child care centres) (Chase-Lansdale et al. 1997). It is also possible that neighbourhood effects on young children operate indirectly (e.g. deprivation, social capital and social cohesion), as mediated through family processes (e.g. maternal mental health, parenting, family dysfunction and the social support received by the mother) (Campbell et al. 2000; Chase-Lansdale et al. 1997; Klebanov et al. 1997; Kohen et al. 2008), since most of their time is spent at home, and their interactions with larger social contexts are determined by and largely experienced through their parents/caregivers.

We focus our investigations on children of preschool years, as this is the stage when vital development occurs in behavioural and cognitive development domains (Phillips and Shonkoff 2000). Early cognitive and socio-emotional development have been shown to predict health and well-being into adolescence and adulthood (Pihlakoski et al. 2006; Spira and Fischel 2005) and differences in socioemotional and behavioural problems between ethnic minority and majority children can be detected in the preschool years (Flink et al. 2013; Jansen et al. 2010). It is important to understand how ethnic density contributes to the development of young children, in order to develop interventions for the preventions of behavioural problems and cognitive deficits among vulnerable groups of children. We aim to estimate the association between ethnic density and early childhood socioemotional and cognitive development, conditional on individual and area-level material deprivation in England and in the US, and as possibly mediated by maternal depression.

## Data and Methods

This study was based on data from two nationally representative birth cohort studies, the UK Millennium Cohort Study (MCS), and the US Early Childhood Longitudinal Study-Birth Cohort (ECLS-B). The MCS and ECLS-B are the most suitable and comparable surveys to examine the study’s aims. Child participants in each study were born around the same year and followed up at similar ages, and both surveys are nationally representative and include data on similar health outcomes and risk factors.

### Millennium Cohort Study

The MCS is a nationally representative cohort survey of 18,819 infants born in the UK between 2000 and 2002. The sample for the baseline cohort included infants who were alive and residing in the UK at 9 months of age, and was drawn from Child Benefit registers (Plewis et al. 2007). Child Benefit claims in the UK cover nearly all children except those who are ineligible because of recent or temporary immigrant status. The MCS sampled from England and Wales, but the Welsh data include very few ethnic minority children and use a different measure of area deprivation. Thus, this analysis uses only the English respondents. The sample was stratified by electoral ward with over sampling of ethnic minority and disadvantaged residential areas. Parents were interviewed at home and the main respondent was usually the natural mother (98%) (Hansen 2014). To enable comparison with the ECLS-B, we used data collected during the third sweep of interviews, when the cohort child was around 5 years of age. During the interview, a range of child outcomes (i.e. cognitive, socioemotional, and behavioural outcomes) were measured, and detailed information was collected on sociodemographic and socioeconomic characteristics. The English analyses included singleton births with natural mothers as main respondent and without missing data on child outcomes and covariates.

### Early Childhood Longitudinal Study-Birth Cohort

The ECLS-B is a nationally representative sample of 10,700 children born in the US in 2001. Children were eligible to be sampled if they were born in the US to mothers aged 15 years or older and if they did not die, move abroad, or get adopted prior to 9 months of age (Bethel et al. 2005). The survey oversampled children who were low birth weight, very low birth weight, twins, American Indian/Alaska Native, Chinese, and other Asian/Pacific Islanders. Home interviews with parents collected information on children’s cognitive, socioemotional, physical development, and sociodemographic circumstances (Snow et al. 2009). We used data from the kindergarten round of data collection, which assessed children at approximately 5 years old when they first entered kindergarten. The US analyses included singleton births with non-missing data on outcomes and the covariates whose biological mothers participated in the survey.

## Individual-Level Measures

### Socioemotional and Behavioural Outcomes

In the MCS, child socio-emotional behaviour was measured when children were aged 5 years using the parent-fill version of the Strengths and Difficulties Questionnaire (SDQ; Goodman 1997). The SDQ is a widely used instrument developed for assessing child socio-emotional behaviour (http://www.sdqinfo.com/), which is a validated tool that has been shown to highly correlate with other measures of health and development (Goodman 1997; Goodman et al. 2000; Goodman and Scott 1999). The SDQ is composed of 25 questions, which cover five domains: conduct problems, hyperactivity and inattention, emotional symptoms, peer problems, and prosocial behaviours. Each question is scored 0 (not at all true), 1 (partly true), or 2 (certainly true), with some questions reversed coded. The SDQ measures two internalising difficulties (emotional symptoms and peer problems) and two externalising difficulties (hyperactivity and conduct problems). The first four domains are summed to construct a total difficulties score as a continuous variable.

The US ECLS-B contained 24 individual items measuring behavioural and socioemotional outcomes, which were not drawn from any one recognised behavioural scale, although many of the items were taken from the Preschool and Kindergarten Behavioural Scales, second edition, and were highly similar to the SDQ items (Waldfogel and Washbrook 2011) in terms of measuring the 5 domains of conduct problems, hyperactivity and inattention, emotional problems, peer problems, and prosocial behaviours. Each ECLS-B item was measured on a five-point scale, from 1 (never) to 5 (very often). For comparability with MCS, we collapsed the two highest and the two lowest responses in order to create a three-point scale.

Since there was an unequal number of behavioural and socioemotional items between MCS and ECLS-B, our analyses utilised mean total difficulties score (across 20 items asked in MCS, across 16 items in ECLS-B) and mean prosocial behaviour score (across 5 items for both surveys). Higher total difficulties scores reflect worse outcomes, whereas higher prosocial behaviour scores reflect better outcomes.

### Cognitive Outcome

The cognitive outcome was measured as picture vocabulary test scores in England and early reading ability in the US. Vocabulary development in the MCS was assessed using a widely validated, age-appropriate test: the naming vocabulary subscale from the British Ability Scale (BAS; Elliott et al. 1996). The BAS naming vocabulary score assesses expressive language and knowledge of naming in English. To remedy the problem of comparability across different sets of items, vocabulary test scores were standardised according to child age, and further adjusted for the difficulty of the items and the ability of the child through the use of item response theory (Rasch 1960, 1961). Higher scores indicate better cognitive outcome.

In the ECLS-B, children’s early reading ability was measured using an ECLS-B designed assessment drawing on existing items from standardised instruments and assessment batteries for preschool- and kindergarten-aged children, such as the Peabody Picture Vocabulary Test and PreLAS^®^ 2000. Our analyses used children’s scale scores on the reading assessment, which reflect the estimated number of items that he/she would have answered correctly, if asked all of the scored questions, based on item response theory (Snow et al. 2009). The two measures used in the MCS and the ECLS-B are not identical but highly similar in reflecting cognitive ability.

### Race/Ethnicity

Racial/ethnic categories were constructed using mother’s reports of her child’s race/ethnicity and were based on census categories within each country. In England, the groups used for analysis were as follows: Indian, Pakistani, Bangladeshi, Black Caribbean (including mixed White and Black Caribbean) and Black African (including mixed White and Black African). For the US, the ethnic groups were as follows: non-Hispanic Black or African American, Hispanic, Asian and American Indian.

Studies of ethnic density do not include the White population because ethnic density theory suggests that experiences unique to racial/ethnic minority populations such as racism and minority status stigma, and their interaction with low SES, are buffered in areas with greater ethnic density. The focus is on the extent to which an increase in own-ethnic density is associated with changes in health outcomes among minority racial/ethnic groups. The analyses are stratified by racial/ethnic group and are not focused on racial/ethnic inequalities. Thus, there is no comparison of racial/ethnic minority and White children.

### Covariates

We included similar socio-demographic characteristics in each country: child’s gender and age, mother’s nativity (born in the US/UK or not), mother’s age at the birth of the cohort child, single parenthood, low birth weight (<2500 g), and whether English was the primary language spoken within household. We also included the following socio-economic factors: mother’s employment status (working full-time, working part-time and unemployed), household income in quintiles and highest maternal educational qualifications. Measures of maternal education were not directly comparable between two countries; for England, maternal educational qualifications were categorised based on UK National Vocational Qualifications ranging from NVQ5 (equivalent to post-graduate qualifications) to NVQ1 (equivalent to D-G grade on General Certificate of Secondary Education in England or some high school education in the US) and no qualification. We included five categories of maternal education: less than O level (ordinary level), O level, A level (advanced level), degree or higher, overseas qualifications or none. For the US, the educational variable was categorised into four levels: less than high school, high school, some college and bachelor’s degree or higher.

We also included measures of maternal depression. In the MCS a measure of maternal depressive symptoms, the six-item Kessler Psychological Distress Scale (K6) was collected at sweep 3. Responses ranged from ‘none of the time’, scored as 0, to ‘all of the time’ scored as 4, resulting in a total K6 score that ranged from 0 to 24. A cut-off score of 13 or higher is commonly used to detect clinical depression and/or anxiety (Kessler et al. 2003). For the US, depressive symptoms were measured by a 12-item abbreviated form of the Centre for Epidemiologic Studies Depression Scale (CES-D; Radloff 1977). The CES-D score ranges from 0 to 36 on a 4-point (ranging from 0 to 3) for each of 12 items. We used a cut-off >9 to represent clinically significant depression, corresponding with the most widely used clinical cut point >15 indicative of depression on the full CES-D (Nord et al. 2005).

## Neighbourhood-Level Predictors

### Ethnic Density

We measured ethnic density based on two geographically analogous scales between the two countries: Medium Super Output Area (MSOA) for England and zip code tabulation areas (ZTCAs) for the US. MSOAs are the middle layer of geographical Output Areas designed by the Office of National Statistics for the collection and publication of small area statistics. In England, they were designed to have similar population sizes and be socially homogenous. A MSOA has a minimum population of 5000, with a mean of 7200, and a ZCTA’s has a median population of 2800 (interquartile range 734–12,945). English MSOAs and US ZCTAs are area definitions commonly used in prior research and have been shown to be comparable geographical units in terms of population size (Iceland et al. 2011).

Ethnic density was measured in both countries as the percentage of residents in the geographical area who were of the same ethnic group, in line with previous studies (e.g. Bécares et al. 2009, 2012a; Pickett et al. 2009). Ethnic density was characterised as a continuous proportion and modelled results report the association for each 10% increase in own ethnic density to facilitate interpretation of results. Because the association of ethnic density with outcomes may not be linear, and because the range of ethnic density varies between ethnic groups (Hutchinson et al. 2009; Mason et al. 2011; Shaw et al. 2010), areas were also categorised as having 0–4.9, 5–29.9, 30–49.9, and >50% own ethnic density (see Appendices 1, 2). These cut-offs were chosen as they were consistent with previous studies (Pickett et al. 2009), and their use enabled us to identify at which level ethnic density was most beneficial or detrimental for the health of ethnic minority children.

### Area Deprivation

For England, we used the Index of Multiple Deprivation (IMD) summary score as a measure of area deprivation. The IMD is a measure of multiple deprivation based on a weighted cumulative model of seven individual domains regarding income deprivation, employment deprivation, health deprivation and disability, education skills and training deprivation, barriers to housing and services, crime and living environment deprivation (Noble et al. 2014). The IMD data were categorised into quintiles for analysis, quintile 1 indicating the most affluent areas, and quintile 5 the most deprived.

In order to aid in comparability of deprivation measures across countries, we constructed an area deprivation indicator for the US based on similar domains as the IMD: % below federal poverty line, % unemployed, % with public assistance, % adults >25 years old without a high school diploma/General Education Development, % adolescents 14–17 enrolled in school, % household overcrowding, % households paying >30% of income on housing. Variables were standardised, and a weighted sum was calculated with weights proportionate to those used in IMD. The final score was categorised into quintiles for analyses.

## Statistical Methods

Sample characteristics are described using means and proportions. In order to understand the overall association between ethnic density and children’s behavioural and cognitive outcomes, and to model the relative contribution of ethnic density and area deprivation to each outcome, we investigated the independent effects of ethnic density in predicting children’s mean total difficulties scores, mean prosocial scores and reading ability scores, after adjustment for socioeconomic and demographic confounders at both individual and area levels. We fitted linear regression models for each outcome in the following sequence. We first fitted crude models (Model 1) which only adjusted for ethnic density. We further adjusted for individual-level covariates (without maternal depression) to examine the role of ethnic density on child outcomes independent of individual-level socioeconomic factors (Model 2).

Based on our hypothesis that maternal mental health may mediate the relationship between ethnic density and children’s outcomes, we tested the relationships between ethnic density and maternal depression, and between maternal depression and children’s outcome scores (see Appendix 3, Table 7). We then additionally adjusted our main models for maternal depression in Model 3 to examine how additional control for this potential mediator impacted our estimates of the relationships of interest between ethnic density and children’s outcome scores. Using the Baron and Kenny approach, mediation may be present if there are significant relationships between the exposure and hypothesised mediator as well as between the hypothesised mediator and outcome, along with attenuation of the main exposure–outcome relationship after adjustment for the hypothesised mediator; this approach relies on the assumption of no unmeasured confounding of these relationships (Baron and Kenny 1986). In fully adjusted models (Model 4), we additionally adjusted for area deprivation and this allowed us to isolate the contributions of ethnic density and area deprivation, in addition to examining the role of area deprivation in masking ethnic density effects, independent of individual-level socioeconomic characteristics.

Due to stratified and clustered design of the MCS and ECLS-B, all analyses were carried out by taking account of the complex survey design and the geographically hierarchical nesting of individuals within small areas in the data, in order to obtain estimates that are nationally representative and to produce unbiased standard errors of these estimates. Analysis was carried out with locally available statistical software by investigators in each country [*svy* commands in Stata version 13 StataCorp. 2013 for MCS; *proc survey* commands in SAS version 9.3 (SAS Institute, Inc., Cary, NC) for ECLS-B]. These packages produce consistent results in complex sample design analysis (Oyeyemi et al. 2010; Siller and Tompkins 2006). Due to confidentiality concerns and data access agreements, some descriptive estimates are suppressed (e.g. cell size lower than 10 in MCS) or rounded (e.g. ECLS-B counts rounded to nearest 50).

## Results

Tables 1 and 2 show children’s behavioural and cognitive outcomes, and the distributions of risk factors according to children’s ethnic groups for England and the US, respectively. In England, Pakistani children and Black Caribbean children had the highest total difficulties scores, whilst in the US Hispanic children were among the highest. Regarding cognitive outcomes, in England Pakistani and Bangladeshi children had the lowest reading vocabulary scores. In the US, Asian children had the highest reading score, whilst American Indian and Hispanic children fared the worst. In both countries, there was substantial heterogeneity in the distributions of risk factors by race/ethnicity. In particular, Pakistani and Bangladeshi children in England, and African American children in the US, were among the most materially disadvantaged.

**Table 1:** Children’s socioemotional, behavioural problems and vocabulary score and the distribution of explanatory factors by race in Millennium Cohort Study, England

	Indian	Pakistani	Bangladeshi	Black Caribbean	Black African
	(*n* = 286)	(*n* = 355)	(*n* = 102)	(*n* = 234)	(*n* = 163)
Outcome					
Average total difficulties score [mean (sd)]	0.36 (0.31)	0.48 (0.38)	0.39 (0.34)	0.41 (0.25)	0.37 (0.30)
Average prosocial score [mean (sd)]	1.73 (0.39)	1.64 (0.49)	1.66 (0.53)	1.69 (0.37)	1.63 (0.48)
BAS vocabulary standard score [mean (sd)]	51.69 (14.89)	43.86 (16.19)	42.70 (15.40)	52.29 (11.11)	49.22 (11.78)
Covariates					
Age in years [mean (sd)]	4.84 (0.47)	4.79 (0.56)	4.82 (0.58)	4.79 (0.46)	4.82 (0.43)
Child is male (%)	51.7	47.9	45.6	47.9	48.8
Low birth weight (<2500 g, %)	12.9	12.5	11.0	11.0	6.2
Mother’s age of birth [mean (sd)]	28.88 (6.52)	26.39 (6.89)	24.97 (5.14)	28.68 (7.21)	30.80 (7.13)
Mother is born in UK (%)	53.2	50.5	13.7	90.5	45.5
Mother’s qualification (%)					
O level or less	22.4	38.8	36.5	16.5	21.0
A level	16.7	23.7	27.6	33.0	14.7
Degree or higher	14.1	14.8	13.8	11.7	8.8
Foreign or none	46.8	22.7	22.0	38.7	55.4
Household income, quintiles					
1 Lowest	15.4	38.8	48.8	37.9	29.5
2	20.9	33.4	27.7	23.2	15.7
3	14.1	17.6	10.7	14.0	21.9
4th + 5th quintiles	49.6	10.2	12.8	24.9	32.9
Mother in employment (%)					
Unemployed	32.2	71.5	70.8	46.6	44.6
Employed	67.8	18.5	29.1	53.4	55.4
Maternal mental health (K6)					
Severe (K6 ≥13, %)	4.9	3.9	^a^	^a^	^a^
Single parenthood (%)	7.7	11.7	^a^	47.1	37.8
Index of multiple deprivation					
1 Least deprived	16.7	61.4	65.5	35.1	45.9
2	24.5	13.9	11.0	31.6	18.2
3	18.9	11.8	11.4	13.6	17.8
4th + 5th quintiles	39.8	12.9	12.1	19.7	18.1
% Own racial/ethnic group [mean (sd)]	14.99 (23.93)	21.81 (29.11)	13.84 (24.27)	6.29 (7.99)	6.92 (8.94)

Figures are percentages that are weighted with overall sampling weights. Sample sizes are unweighted. The use of these data does not imply the endorsement of the data owner or the UK Data Service at the UK Data Archive in relation to the interpretation or analysis of the data. This work uses research datasets which may not exactly reproduce National Statistics aggregates

*BAS* British Ability Scales, *A level* advanced level, *O level* ordinary level

^a^Sample sizes smaller than 10, estimates cannot be displayed due to confidential concerns

**Table 2:** Children’s socioemotional, behavioural problems and reading ability and the distribution of explanatory factors by race in Early Childhood Longitudinal Study-Birth Cohort, the US

	African-American (*n* = 780)	Hispanic (*n* = 950)	Asian (*n* = 750)	American Indian (*n* = 200)
Outcome				
Average total difficulties score [mean (sd)^a^]	0.4 (0.3)	0.5 (0.3)	0.4 (0.3)	0.44 (0.3)
Average prosocial score [mean (sd)^b^]	1.5 (0.5)	1.5 (0.5)	1.5 (0.5)	1.5 (0.5)
Reading scale score [mean (sd)^c^]	41.5 (14.0)	38.9 (14.1)	52.8 (15.2)	38.3 (14.2)
Covariates				
Child’s age in years [mean (sd)]	5.7 (0.4)	5.6 (0.4)	5.6 (0.4)	5.6 (0.3)
Child is male (%)	51.8	53.1	52.8	57.6
Low birth weight (<2500 g, %)	10.9	5.9	6.7	2.3
Mother’s age at birth [mean (sd)]	25.6 (6.3)	26.2 (6.1)	29.9 (5.6)	24.9 (5.8)
Language spoken at home is primarily English (%)	95.8	34.9	38.6	98.3
Mother is native-born (%)	91.9	40.0	14.8	99.6
Household income, quintiles				
1 Lowest	41.5	30.0	4.6	26.0
2	25.9	33.2	12.5	41.9
3	15.0	19.8	20.1	16.6
4	9.3	9.7	19.8	11.5
5 Highest	8.3	7.4	43.0	3.9
Highest employment level in household (%)				
Full-time	67.8	82.6	91.2	60.7
Part-time	10.5	7.4	3.4	24.2
Not employed	21.7	10.0	5.4	15.1
Mother’s educational attainment (%)				
Less than high school	16.6	35.1	4.1	15.8
Completed high school	41.8	36.2	21.5	37.4
Some college	29.9	20.8	16.2	35.1
Bachelor’s degree or higher	11.7	7.9	58.3	11.8
Single parenthood (%)	58.0	22.1	8.2	41.5
Multiple deprivation index				
1 Least deprived	5.8	7.7	23.0	2.4
2	10.2	11.7	24.7	16.4
3	15.2	15.9	20.2	18.3
4	26.9	22.6	17.1	33.5
5 Most deprived	41.8	42.0	14.9	29.3
Maternal mental health				
Clinically significant symptoms (score >9, %)	24.6	13.2	12.4	15.5
% Own racial/ethnic group [mean (sd)]	42.9 (29.4)	39.9 (26.6)	14.2 (16.5)	14.5 (25.6)

Figures are percentages that are weighted with overall sampling weights. Sample sizes are unweighted. Unweighted sample sizes are rounded to the nearest 50

^a^For the total difficulties score, there were fewer than 50 missing scores

^b^For the prosocial score, there were 100 missing scores

^c^For the reading scale outcome, there were 50 missing scores

### Ethnic Density Effects Across Countries

There was variation in ethnic density between ethnic groups in England (Table 1) and in the US (Table 2). African American and Hispanic children tended to live in areas where around 40% of residents were of the same race/ethnicity. In England, Pakistani children experienced the highest levels of own ethnic density (around 22%); Black African (around 6%) and Black Caribbean (around 7%) groups had the lowest levels of ethnic density. These patterns reflect differences in the recency of settlement in England in the US across different racial/ethnic minority groups, and the histories of geographical settlement described in the Introduction section.

Tables 3 and 4 present the associations between a 10% increase in ethnic density and socioemotional and cognitive development for children in the US and England, respectively. We have used this modelling strategy in our previous work analyses, and it generally serves as a standard in this literature. In crude models, we found a detrimental effect of higher ethnic density on socioemotional development for all racial/ethnic groups except for the Asian group in the US. We found a trend for a detrimental effect of own ethnic density and socioemotional development for American Indian children whereby when American Indian ethnic density increased by 10%, the mean total difficulties scores increased by 0.021 (*p* < 0.05; Table 4, Model 1). Upon adjustment for individual-level confounders, the detrimental effect for American Indian children weakened and became insignificant, and further adjustment for area deprivation changed the direction of ethnic density effect, becoming protective, although not significant (Table 4, Model 4). Maternal mental depression was a significant predictor of child outcomes but it did not meaningfully attenuate the association between own ethnic density and child outcomes in the US (Table 4, Models 2 and 3).

**Table 3:** Association between ethnic density (10% increase) and children’s behavioural and cognitive outcomes in Millennium Cohort Study, England

	Model 1			Model 2			Model 3			Model 4		
	*β*	(95% CI)		*β*	(95% CI)		*β*	(95% CI)		*β*	(95% CI)	
Mean total difficulties scores												
Indian												
Ethnic density	0.016	−0.01	0.04	0.005	−0.016	0.027	0.005	−0.002	0.003	−0.004	−0.002	0.02
Maternal depression							0.174	−0.06	0.41	0.19	−0.03	0.40
Pakistani												
Ethnic density	−0.006	−0.02	0.01	−0.014*	−0.03	−0.000	−0.013	−0.03	0.0001	−0.01	−0.03	0.004
Maternal depression							0.26*	0.06	0.46	0.27*	0.06	0.47
Bangladeshi												
Ethnic density	0.007	−0.02	0.03	0.005	−0.015	0.025	0.006	−0.02	0.03	0.009	−0.01	0.03
Maternal depression							0.068	−0.012	0.26	0.06	−0.13	0.24
Black Caribbean												
Ethnic density	0.07**	0.02	0.12	0.05*	0.007	0.09	0.046*	0.005	0.09	0.03	−0.02	0.08
Maternal depression							0.16***	0.07	0.25	0.15***	0.06	0.23
Black African												
Ethnic density	0.001	−0.06	0.06	0.009	−0.06	0.08	0.008	−0.06	0.08	−0.014	−0.09	0.06
Maternal depression							0.08	−0.28	0.44	0.08	−0.26	0.41
Mean behavioural scores												
Indian												
Ethnic density	0.003	−0.02	0.01	0.002	−0.02	0.015	−0.001	−0.17	0.014	0.007	−0.01	0.002
Maternal depression							−0.27	−0.57	0.03	−0.29*	−0.57	−0.02
Pakistani												
Ethnic density	−0.018	−0.04	0.01	−0.015	−0.04	0.008	−0.016	−0.04	0.01	−0.02	−0.05	0.01
Maternal depression							−0.23*	−0.39	−0.07	−0.24**	−0.40	−0.02
Bangladeshi												
Ethnic density	0.016	−0.03	0.06	0.05*	0.006	0.10	0.05*	0.005	0.09	0.03	−0.01	0.08
Maternal depression							−0.16	−0.55	0.23	−0.22	−0.60	0.16
Black Caribbean												
Ethnic density	−0.03	−0.13	0.07	0.01	−0.05	0.07	0.014	−0.05	0.08	0.04	−0.04	0.13
Maternal depression							−0.12	−0.26	0.02	−0.11	−0.25	0.02
Black African												
Ethnic density	−0.024	−0.08	0.04	−0.04	−0.09	0.02	−0.035	−0.09	0.02	−0.03	−0.10	0.04
Maternal depression							−0.22	−0.60	0.16	0.20	−0.59	0.18
Naming vocabulary scores												
Indian												
Ethnic density	−0.67*	−1.25	−0.09	0.016	−0.59	0.62	0.02	−0.58	0.61	0.15	−0.63	0.94
Maternal depression							−0.92	−9.84	8.00	−5.53	−11.87	0.81
Pakistani												
Ethnic density	−1.08**	−1.80	−0.37	−0.42	−1.00	0.16	−0.45	−1.00	0.10	−0.43	−1.10	0.25
Maternal depression							−7.30**	−11.80	−2.80	−7.00**	−11.56	−2.45
Bangladeshi												
Ethnic density	−1.54*	−2.81	−0.28	−0.75	−1.96	0.45	−0.51	−1.41	0.40	−1.17*	−2.18	−0.16
Maternal depression							13.46*	3.25	23.67	12.25*	1.37	23.12
Black Caribbean												
Ethnic density	−1.12	−3.01	0.77	0.19	−0.20	0.23	0.33	−1.90	2.56	0.97	−1.15	3.09
Maternal depression							−6.89***	−10.77	−3.01	−6.38***	−10.15	−2.60
Black African												
Ethnic density	−2.43*	−4.35	−0.52	−1.21	−2.75	0.33	−1.23	−2.69	0.23	−1.71	−3.56	0.12
Maternal depression							7.27	−1.09	15.62	7.54*	0.11	14.96

*Model 1* adjusts for own ethnic density, *Model 2* additionally adjusts for child’s age, sex, low birth weight, maternal age at birth, English as primary household language, maternal nativity, household size, single household income (quintiles), maternal employment status in household, maternal educational attainment based on Model 1, *Model 3* additionally adjusts for maternal depression based on Model 2, *Model 4* additionally adjusts for multiple deprivation index (quintiles) based on Model 3

* *p* < 0.05, ** *p* < 0.01, *** *p* < 0.001

**Table 4:** Association between ethnic density (10% increase) and children’s behavioural and cognitive outcomes in the Early Childhood Longitudinal Study-Birth Cohort, the US

	Model 1			Model 2			Model 3			Model 4		
	*β*	(95% CI)		*β*	(95% CI)		*β*	(95% CI)		*β*	(95% CI)	
Mean total difficulties scores												
African-American												
Ethnic density	0.006	−0.002	0.015	0.0002	−0.009	0.009	0.001	−0.008	0.009	−0.003	−0.014	0.009
Maternal depression							0.138***	0.082	0.193	0.137***	0.080	0.193
Hispanic												
Ethnic density	0.003	−0.004	0.009	−0.005	−0.012	0.002	−0.005	−0.012	0.003	−0.005	−0.014	0.004
Maternal depression							0.152***	0.093	0.211	0.153***	0.093	0.212
Asian												
Ethnic density	−0.002	−0.017	0.014	0.002	−0.013	0.017	0.001	−0.014	0.016	0.003	−0.013	0.018
Maternal depression							0.114**	0.035	0.192	0.117**	0.041	0.193
American Indian												
Ethnic density	0.021*	0.002	0.040	0.015	−0.008	0.037	0.015	−0.007	0.037	−0.003	−0.032	0.026
Maternal depression							−0.018	−0.141	0.106	−0.002	−0.127	0.123
Mean prosocial behavioural scores												
African-American												
Ethnic density	−0.009	−0.023	0.004	−0.003	−0.019	0.012	−0.003	−0.019	0.012	−0.004	−0.021	0.013
Maternal depression							−0.040	−0.147	0.067	−0.039	−0.145	0.066
Hispanic												
Ethnic density	−0.008	−0.025	0.009	−0.001	−0.018	0.016	−0.001	−0.018	0.016	0.001	−0.018	0.021
Maternal depression							0.045	−0.060	0.151	0.051	−0.051	0.153
Asian												
Ethnic density	−0.023	−0.054	0.008	−0.021	−0.051	0.008	−0.021	−0.051	0.009	−0.026	−0.057	0.006
Maternal depression							−0.014	−0.134	0.105	−0.021	−0.140	0.099
American Indian												
Ethnic density	−0.001	−0.029	0.027	0.011	−0.013	0.036	0.012	−0.013	0.037	0.039*	0.008	0.071
Maternal depression							−0.018	−0.313	0.278	−0.050	−0.345	0.246
Early reading scale scores												
African-American												
Ethnic density	−0.530*	−0.977	−0.084	−0.098	−0.468	0.272	−0.104	−0.470	0.262	0.016	−0.389	0.420
Maternal depression							−0.992	−3.261	1.278	−1.049	−3.341	1.243
Hispanic												
Ethnic density	−0.470*	−0.863	−0.078	−0.037	−0.408	0.335	−0.037	−0.407	0.332	0.095	−0.397	0.587
Maternal depression							−0.335	−3.299	2.629	−0.423	−3.391	2.544
Asian												
Ethnic density	0.260	−0.696	1.216	0.462	−0.313	1.236	0.457	−0.315	1.228	0.422	−0.437	1.281
Maternal depression							1.898	−1.721	5.518	1.872	−1.666	5.409
American Indian												
Ethnic density	−0.561	−1.670	0.548	−0.126	−0.836	0.584	−0.134	−0.845	0.576	0.080	−0.839	0.999
Maternal depression							0.686	−3.408	4.781	1.115	−3.029	5.259

*Model 1* adjusts for own ethnic density, *Model 2* additionally adjusts for child’s age, sex, low birth weight, maternal age at birth, English as primary household language, maternal nativity, household size, single household income (quintiles), highest employment status in household, maternal educational attainment based on Model 1, *Model 3* additionally adjusts for maternal depression based on Model 2, *Model 4* additionally adjusts for multiple deprivation index (quintiles) based on Model 3

* *p* < 0.05, ** *p* < 0.01, *** *p* < 0.001

Results for English Black Caribbean children showed a detrimental effect of Caribbean ethnic density on total difficulties scores, independent of individual and household characteristics (Table 3, Model 3), whereby as Caribbean ethnic density increased by 10%, total difficulties scores increased by 0.046 (*p* < 0.05). This detrimental effect weakened and became insignificant after adjustment for area deprivation (Table 3, Model 4). For other racial/ethnic groups in England, such as Indian, Pakistani and Black African, own ethnic density showed a non-statistically significant protective effect on total difficulties scores (Table 3, Model 4).

With regard to prosocial behaviour scores, results of crude models show a detrimental effect of ethnic density for all racial/ethnic groups in the US, although all these associations were not statistically significant (Table 4, Model 1). Upon adjustment for individual-level covariates and area-level deprivation, the detrimental ethnic density effect for American Indian children reversed, becoming protective and statistically significant (*p* < 0.05) so that an increase of 10% in American Indian density was associated with an increase of prosocial score by 0.039 (95% CI 0.008, 0.071; Table 4, Model 4).

In England, we found a statistically significant and protective association for Bangladeshi children (Table 3, Models 1–3). However, on further adjustment for area deprivation, this protective association became smaller and statistically insignificant (Table 3, Model 4). The association between ethnic density and children’s socioemotional outcomes was attenuated with adjustment for maternal depression, particularly among Pakistani and Black Caribbean children.

With regard to cognitive outcomes, Table 4 shows that there were no significant associations between ethnic density and reading ability scores for children in the US in the fully adjusted models. There was a negative association between own ethnic density and reading ability scores for all racial/ethnic groups except for Asian children in crude models, although the associations were only significant for African American and Hispanic children (Table 4, Model 1). The direction of ethnic density effects remained unchanged after adjustment for individual-level covariates for all racial/ethnic groups (Table 4, Models 2 and 3). After further adjustment for area deprivation (Table 4, Model 4), the direction of ethnic density effects was reversed from a detrimental to a protective effect for Hispanic and American Indian children. Among Asian children, a protective association persisted across the three models, although the protective association weakened after controlling for area deprivation. Ethnic density effects in England showed a different picture.

We found detrimental associations between own ethnic density and cognitive outcomes for all racial/ethnic groups in England in the crude models (Table 3, Model 1). However, all these negative effects weakened and became statistically insignificant upon adjustment for individual and household characteristics, and some effects reversed directions becoming protective for Indian and Black Caribbean children (Table 3, Model 3). Adjustment for area deprivation tended to strengthen negative ethnic density effects among Bangladeshi and Black African children (Table 3, Model 4). For example, an increase of 10% Bangladeshi density was associated with a decrease of 1.17 in children’s naming vocabulary scores (*p* < 0.05).

Results from models operationalizing ethnic density using categorical variables are largely but not completely consistent with main results (Appendix Tables 5, 6). For example, total difficulty scores are modestly lower for Bangladeshi children with greater than 5% own-group ethnic density when compared to children in areas with <5% own-group, and Pakistani children in higher-density neighbourhoods have higher prosocial behaviour scores than children in <5% own-group areas.

## Discussion and Conclusion

We used two nationally representative cohort studies to investigate the differences and similarities in ethnic density effects on behavioural and cognitive outcomes among young children in the US and England. We found substantial heterogeneity in ethnic density effects on child outcomes within and between two countries. In the US, an increase in ethnic density was associated with improved prosocial behavioural outcomes among American Indian children, although the sample size for this group is small. In England, increased ethnic density was associated with increased total difficulties scores among Black Caribbean children and improved social behavioural outcomes for Bangladeshi children, although in both cases the associations lost statistical significance after adjustment for area deprivation. In terms of cognitive outcomes, increased own ethnic density was associated with reduced cognitive scores for most ethnic groups in England, especially for Bangladeshi children, the only group where the effect remained statistically significant with the full set of controls. In the US, increased own ethnic density showed mixed and non-significant effects on cognitive scores for all racial/ethnic groups except for a negative effect for African American children before additional controls were added. Asian children showed a consistent positive effect but with large standard errors.

The finding for American Indian children stands out as notable because few studies have included these children. While the ECLS sample of American Indian children is numerically small, it is one of the largest population-based representative samples of this group, and thus worth exploring with due attention to limitations of inference from this small sample. The history of residential settlement for American Indian communities is complex with some tribes forcibly relocated to reservations far from traditional lands (e.g. Cherokee), and others forcibly constrained to traditional lands (e.g. Navajo). Beyond residence on reservations, there is a sizeable and growing population of urban American Indians (Baldwin et al. 2002). In the ECLS-B, the majority of American Indian respondents were from rural areas consistent with residence on reservations. This unique historical experience of isolation and area deprivation for American Indians—including federal government efforts to eradicate traditional language and cultural practices, forced eradication of traditional language, illegalised spiritual practices, and forced removal from their lands (Duran and Duran 1995)—seems particularly poised to lead to poor child outcomes. It has been suggested that traditional practices, traditional spirituality, and cultural identity are positively related to prosocial behaviours and self-efficacy of children (Whitbeck 2006; Whitbeck et al. 2001; Zimmerman et al. 1998). Future research with larger sample sizes and correspondingly greater statistical power could better document the ethnic density effect for this group.

In general, the magnitude of the associations observed here was small, and we found relatively larger associations of own ethnic density on cognitive outcomes in England compared to the US. One possible explanation for this is related to the measures for cognitive outcomes between two studies, which are not completely comparable. The MCS assessed expressive vocabulary by asking the cohort child to name out loud the object shown in a single picture. This differs from the receptive vocabulary that was assessed in the ECLS-B, in which the child is shown pictures and asked to identify the one that best represents the meaning of the word read by the interviewer (Washbrook et al. 2012). It is also possible that the national context and historical patterns of residential settlement in England bestows more protective effects on ethnic density than in the US. Several studies have reported that living in neighbourhoods with higher immigrant concentrations may be associated with cognitive development and educational achievement among immigrant children (Georgiades et al. 2007; Jensen and Rasmussen 2011). Our findings considered but did not model the variation in immigration history among racial/ethnic groups as well as between two comparative countries.

We investigated one potential family process mechanism. We hypothesised that area-based ethnic density might affect children’s socioeconomic and cognitive development through maternal mental health. If residence in an ethnic enclave provided social support and culturally appropriate role modelling for mothers as the primary caregiver, children in these families could fare better. While we do find evidence for an independent effect of maternal depression on outcomes, it does not appear that maternal depression meaningfully mediates most ethnic density–child outcomes associations. Ethnic/racial and cultural variations in parenting practices (Julian et al. 1994), child-care quality (Burchinal et al. 2000) and expectations for educational achievement (Goyette and Xie 1999; Kao and Thompson 2003) have been well documented, which may contribute to different ethnic density effects on child outcomes in this study. There are, however, some important and unmeasured characteristics of parents (for example, parental preferences, concerns about child development and local supply conditions) that may motivate them to choose better or worse neighbourhoods (Duncan et al. 1997). For example, higher-SES racial/ethnic groups who are more concerned about child development may choose to live in affluent neighbourhoods where racial/ethnic groups are less concentrated. Therefore, our estimates are prone to selection bias arising from non-random parental selection of neighbourhoods (Duncan and Raudenbush 1999).

This study is not without limitations. First, the cross-sectional analysis limits our ability to make causal claims due to lack of the temporal ordering of socioeconomic characteristics, ethnic density and child behavioural and cognitive outcomes. Because of this cross-sectional design, we were not able to identify how the racial/ethnic composition changes over time in children’s residential areas, which can be important for child behavioural and cognitive outcomes. Second, in each country context, we assumed that the measurement of ethnic density aggregated to specific geographic areal units (ZCTAs in the US and Medium Super Outer Areas in England) approximated the relevant social context of children. However, it is possible that an ethnic density effect is only apparent at smaller or larger spatial scales. Third, child outcome measures in this study may introduce some bias into our analyses. The measure of child socio-emotional behaviour used here was reported primarily by the child’s mother, which may be subject to report bias. It is possible that mothers who are psychologically distressed may be more likely to report higher behavioural scores of their children than non-depressed mothers (Gartstein et al. 2009).

The measurement of racial/ethnic groups may also introduce bias to our results. For example, the Asian racial group in the US is a combination of East Asian (i.e. Chinese, Japanese), South Asian (i.e. Indian) and other Asians. This aggregation of ethnic groups may obscure differences in socioeconomic and cultural profiles and child behavioural and cognitive development. Along those lines, we were also unable to account for an additional layer of potential diversity by religion. Religious affiliation may shape experiences of racism and minority status stigma in ways that remain unexplored in large population representative data sets due to sample sizes.

This study only documents the influence of ethnic density on young children’s behavioural and cognitive outcomes, and apart from examination of maternal depression, it fails to gain insight into the mechanisms by which ethnic density affect child outcomes. Future studies may benefit from exploring additional family processes (for example, maternal social support, child-rearing practices, parent–child warmth/interaction) (Kohen et al. 2008; Sampson 1992) and neighbourhood social processes (for example, physical and social disorder as well as parent report of social cohesion) (Kohen et al. 2002) in order to take important further steps of developing effective preventive interventions to foster the healthy development of racial/ethnic children.

This paper has benefitted greatly from the availability of two data sets parallel in historical timing and design and their comprehensive measurement of health and developmental outcomes and social determinants of health. The MCS and the ECLS-B are some of the most robust and extensive studies on racial/ethnic minority children, but despite their relatively large total sample sizes and, in the case of ECLS-B, oversampling of some ethnic groups, the sample sizes of some racial/ethnic minority groups are small, which may have resulted in limited statistical power. Despite these limitations, the present study provides novel information on ethnic density effects among racial/ethnic minority children in the US and in England, and shows that benefits and detriments of living amongst other racial/ethnic minority people are dependent on the wider context.

## Appendix 1

See Table 5.

**Table 5:** Association between ethnic density and children’s behavioural and cognitive outcomes in Millennium Cohort Study, England

	Model 1			Model 2			Model 3		
	*β*	(95% CI)		*β*	(95% CI)		*β*	(95% CI)	
Mean total difficulties scores									
Indian									
0–4.99% (Reference)									
5–29.99%	−0.20	−0.40	0.01	−0.22*	−0.42	−0.03	−0.20	−0.39	0.0001
30–49.99%	−0.28	−0.57	0.004	0.38**	−0.61	−0.15	−0.28*	−0.52	−0.04
≥50%	−0.19***	−0.23	−0.14	−0.26***	−0.38	−1.33	−0.20**	−0.34	−0.06
Pakistani									
0–4.99% (Reference)									
5–29.99%	−0.17*	−0.32	−0.02	−0.10	−0.28	0.08	−0.14	−0.32	0.03
30–49.99%	0.20*	0.04	0.35	0.18	−0.001	0.37	0.20*	0.01	0.39
≥50%	0.13***	0.09	0.17	0.09	−0.07	0.24	0.08	−0.8	0.24
Bangladeshi									
0–4.99% (Reference)									
5–29.99%	0.15	−0.29	0.59	−0.18	−0.45	0.09	−0.24*	−0.45	−0.04
30–49.99%	0.07	−0.05	0.20	−0.22	−0.45	0.01	−0.32*	−0.56	−0.08
≥50%	0.03	−0.02	0.07	−0.19	−0.38	0.002	−0.27**	−0.44	−0.11
Black Caribbean									
0–4.99% (Reference)									
5–29.99%	−0.07	−0.21	0.06	−0.03	−2.01	0.14	−0.08	−0.29	0.12
30–49.99%	−0.09	−0.27	0.09	−0.12	−0.30	0.06	−0.17	−0.40	0.05
≥50%	0.03	−0.01	0.07	0.01	−0.13	0.15	−0.08	−0.32	0.16
Black African									
0–4.99% (Reference)									
5–29.99%	−0.12	−0.38	0.14	−0.11	−0.32	0.11	−0.06	−0.28	0.15
30–49.99%	−0.26	−0.62	0.09	−0.20	−0.5	0.09	−0.16	−0.47	0.15
≥50%	−0.20	−0.43	0.03	−0.15	−0.31	0.01	−0.14	−0.30	0.03
Mean prosocial behavioural scores									
Indian									
0–4.99% (Reference)									
5–29.99%	−0.34*	−0.64	−0.03	−0.25*	−0.49	−0.02	−0.28*	−0.51	−0.05
30–49.99%	−0.24	−0.60	0.12	−0.07	−0.39	0.24	0.15	−0.49	0.19
≥50%	−0.27***	−0.32	−0.23	−0.17**	−0.28	−0.06	−0.2**	−0.34	−0.06
Pakistani									
0–4.99% (Reference)									
5–29.99%	0.54***	0.44	0.64	0.48***	0.32	0.64	0.46***	0.27	0.65
30–49.99%	0.34***	0.19	0.50	0.41**	0.17	0.64	0.42**	0.18	0.66
≥50%	0.23***	0.19	0.27	0.26**	0.09	0.44	0.26**	0.09	0.44
Bangladeshi									
0–4.99% (Reference)									
5–29.99%	−0.27*	−0.52	−0.02	−0.08	−0.49	0.34	−0.14	−0.70	0.42
30–49.99%	−0.40	−1.07	0.27	−0.10	−0.83	0.63	−0.02	−0.74	0.70
≥50%	−0.35***	−0.46	−0.23	−0.07	−0.32	0.18	−0.27	−0.77	0.24
Black Caribbean									
0–4.99% (Reference)									
5–29.99%	0.26***	−0.4	−0.12	−0.21	−0.46	0.33	−0.21	−0.47	0.06
30–49.99%	0.31**	−0.54	−0.09	−0.29*	−0.50	−0.07	−0.28*	−0.54	−0.02
≥50%	0.32***	−0.40	−0.25	−0.30**	−0.49	−0.11	−0.29*	−0.54	−0.04
Black African									
0–4.99% (Reference)									
5–29.99%	−0.28**	−0.48	−0.08	−0.44**	−0.71	−0.17	−0.55***	−0.80	−0.3
30–49.99%	−0.20	−0.49	0.09	−0.35	−0.73	0.03	−0.49*	−0.91	−0.08
≥50%	−0.26**	−0.41	−0.10	−0.33**	−0.56	−0.10	−0.38**	−0.60	−0.16
Naming vocabulary scores									
Indian									
0–4.99% (Reference)									
5–29.99%	12.36***	7.18	17.54	12.00**	4.38	19.61	10.35**	2.63	18.06
30–49.99%	3.45	−4.36	11.26	8.35	−3.69	20.07	4.31	−8.71	17.33
≥50%	4.26***	2.22	6.30	5.67	−0.09	11.43	3.39	−3.16	9.94
Pakistani									
0–4.99% (Reference)									
5–29.99%	24.34***	14.99	33.69	16.37**	7.04	25.7	15.97**	5.93	26.12
30–49.99%	17.34***	10.61	24.07	11.98**	4.05	19.91	12.04**	3.74	20.35
≥50%	2.90**	1.23	4.57	1.84	−5.37	9.06	2.01	−5.18	9.20
Bangladeshi									
0–4.99% (Reference)									
5–29.99%	15.47**	4.65	26.29	25.63***	13.67	37.59	24.29**	10.34	38.24
30–49.99%	6.97	−6.26	20.2	17.60**	6.48	28.72	18.98*	4.48	33.49
≥50%	6.02***	3.11	8.92	17.04***	10.09	24.00	12.64*	0.78	24.50
Black Caribbean									
0–4.99% (Reference)									
5–29.99%	8.55***	5.56	11.54	6.44	−1.50	14.39	5.22	−3.66	14.09
30–49.99%	6.39*	1.13	11.65	1.52	−8.51	11.55	0.11	−10.88	11.11
≥50%	0.15	−1.33	1.63	−2.04	−9.69	5.61	−3.30	−12.90	6.31
Black African									
0–4.99% (Reference)									
5–29.99%	2.77	−7.67	13.21	1.19	−9.37	11.77	0.53	−9.60	10.66
30–49.99%	−6.46	−15.10	2.18	−5.25	−13.67	3.16	−6.46	−15.44	2.51
≥50%	−4.43	−11.48	2.63	−2.83	−10.29	4.62	−3.66	−10.78	3.46

*Model 2* adjusts for child’s age, sex, low birth weight, maternal age at birth, English as primary household language, maternal nativity, household size, single parenthood, maternal depression, household income (quintiles), maternal employment status in household, maternal educational attainment, *Model 3* additionally adjusts for multiple deprivation index (quintiles)

* *p* < 0.05, ** *p* < 0.01, *** *p* < 0.001

## Appendix 2

See Table 6.

**Table 6:** Association between ethnic density and children’s behavioural and cognitive outcomes in the Early Childhood Longitudinal Study-Birth Cohort, the US

	Model 1			Model 2			Model 3		
	*β*	(95% CI)		*β*	(95% CI)		*β*	(95% CI)	
Mean total difficulties scores									
African-American									
0–4.99% (Reference)									
5–29.99%	0.018	−0.066	0.101	−0.015	−0.084	0.054	−0.026	−0.097	0.045
30–49.99%	0.058	−0.035	0.150	0.004	−0.077	0.086	−0.020	−0.114	0.073
≥50%	0.063	−0.020	0.146	0.006	−0.071	0.082	−0.021	−0.120	0.077
Hispanic									
0–4.99% (Reference)									
5–29.99%	0.030	−0.038	0.115	−0.010	−0.080	0.061	−0.028	−0.101	0.045
30–49.99%	0.052	−0.031	0.136	−0.025	−0.100	0.051	−0.050	−0.135	0.036
≥50%	0.058	−0.014	0.129	−0.023	−0.097	0.051	−0.035	−0.121	0.052
Asian									
0–4.99% (Reference)									
5–29.99%	0.049	−0.006	0.104	0.067*	0.010	0.123	0.072*	0.017	0.127
30–49.99%	0.055	−0.037	0.147	0.072	−0.017	0.161	0.072	−0.017	0.161
≥50%	−0.032	−0.123	0.060	−0.013	−0.102	0.077	−0.005	−0.095	0.085
American Indian									
0–4.99% (Reference)									
5–29.99%	0.128*	0.015	0.240	0.107	0.011	0.204	0.083	0.000	0.166
30–49.99%	0.149	−0.061	0.359	0.153	−0.044	0.351	0.168	−0.034	0.371
≥50%	0.162*	0.011	0.313	0.106	−0.058	0.271	−0.040	−0.256	0.177
Mean prosocial behavioural scores									
African-American									
0–4.99% (Reference)									
5–29.99%	0.029	−0.157	0.214	0.068	−0.101	0.236	0.068	−0.098	0.234
30–49.99%	−0.001	−0.182	0.181	0.073	−0.099	0.245	0.075	−0.105	0.256
≥50%	−0.037	−0.220	0.146	0.031	−0.148	0.211	0.033	−0.154	0.220
Hispanic									
0–4.99% (Reference)									
5–29.99%	0.040	−0.108	0.188	0.093	−0.053	0.239	0.062	−0.067	0.190
30–49.99%	−0.059	−0.225	0.107	0.010	−0.160	0.181	−0.017	−0.161	0.127
≥50%	−0.031	−0.186	0.123	0.051	−0.107	0.209	0.030	−0.113	0.174
Asian									
0–4.99% (Reference)									
5–29.99%	−0.090	−0.193	0.013	−0.101*	−0.195	−0.007	−0.112*	−0.206	−0.018
30–49.99%	−0.127	−0.297	0.043	−0.146	−0.309	0.017	−0.154	−0.323	0.015
≥50%	−0.216	−0.495	0.062	−0.194	−0.445	0.056	−0.220	−0.479	0.039
American Indian									
0–4.99% (Reference)									
5–29.99%	−0.232	−0.533	0.069	−0.189	−0.408	0.031	−0.160	−0.206	0.02
30–49.99%	0.220	−0.140	0.580	0.307	−0.094	0.709	0.231	−0.217	0.680
≥50%	−0.040	−0.267	0.186	0.063	−0.122	0.247	0.266	0.022	0.510
Early reading scale scores									
African-American									
0–4.99% (Reference)									
5–29.99%	−0.546	−6.320	5.227	1.563	−3.328	6.454	1.778	−3.490	7.046
30–49.99%	−1.338	−6.906	4.231	2.039	−2.634	6.712	2.883	−2.704	8.470
≥50%	−3.849	−9.461	1.764	0.536	−4.151	5.223	1.698	−3.840	7.235
Hispanic									
0–4.99% (Reference)									
5–29.99%	−6.461**	−10.551	−2.371	−4.693**	−7.990	−1.397	−2.805	−6.769	1.159
30–49.99%	−8.142***	−12.504	−3.779	−4.250*	−8.183	−0.317	−1.209	−6.121	3.702
≥50%	−7.435***	−11.410	−3.460	−3.724*	−7.256	−0.192	−1.216	−5.998	3.556
Asian									
0–4.99% (Reference)									
5–29.99%	−0.047	−3.962	3.869	0.637	−3.011	4.285	0.534	−3.128	4.196
30–49.99%	2.300	−3.791	8.391	1.475	−3.891	6.840	1.530	−4.126	7.186
≥50%	0.532	−5.737	6.801	2.331	−2.770	7.432	2.028	−3.516	7.572
American Indian									
0–4.99% (Reference)									
5–29.99%	3.701	−5.669	13.070	2.002	−3.332	7.335	2.834	−2.411	8.078
30–49.99%	−0.090	−10.307	10.128	−0.892	−8.382	6.598	3.409	−3.637	10.455
≥50%	−4.185	−12.257	3.886	−0.440	−5.628	4.747	1.011	−5.395	7.417

*Model 2* adjusts for child’s age, sex, low birth weight, maternal age at birth, English as primary household language, maternal nativity, household size, single parenthood, maternal depression, household income (quintiles), highest employment status in household, maternal educational attainment, *Model 3* additionally adjusts for multiple deprivation index (quintiles)

* *p* < 0.05, ** *p* < 0.01, *** *p* < 0.001

## Appendix 3

See Table 7.

**Table 7:** Associations between own ethnic density (10% increase) and maternal depression in England (Millennium Cohort Study), and the US (Early Childhood Longitudinal Study-Birth Cohort)

	Maternal depression		
	Odds ratios	95% CI	
Ethnic density: England			
Indian	1.01	0.99	1.03
Pakistani	1.00	0.97	0.88
Bangladeshi	0.99	0.93	1.04
Black Caribbean	1.06	0.99	1.13
Black African	1.01	0.93	0.75
Ethnic density: the US			
African American	0.99	0.91	1.09
Hispanic	0.94	0.84	1.04
Asian	1.05	0.88	1.25
American Indian	1.14	0.88	1.46

All models fully adjusted for child’s age, sex, low birth weight, maternal age at birth, English as primary household language, maternal nativity, household size, single parenthood, maternal depression, household income (quintiles), employment status in household, maternal educational attainment, multiple deprivation index (quintiles)

* *p* < 0.05, ** *p* < 0.01, *** *p* < 0.001

## Appendix 4

See Table 8.

**Table 8:** Description of area-level deprivation by level of own ethnic density Millennium Cohort Study, England

Ethnic groups	Multiple Deprivation Index quintiles									
	Quintile 1 (least deprived)		Quintile 2		Quintile 3		Quintile 4		Quintile 5 (most deprived)	
	*n*	Weighted%	*n*	Weighted%	*n*	Weighted%	*n*	Weighted%	*n*	Weighted%
Indian										
0–4.99%	0	0	0	0	^a^	^a^	0	0	0	0
5–29.99%	0	0	^a^	^a^	^a^	^a^	^a^	^a^	0	0
30–49.99%	0	0	^a^	^a^	0	0	^a^	^a^	0	0
≥50%	79	18.2	79	24.5	47	18.9	34	19.5	35	20.3
Pakistani										
0–4.99%	^a^	^a^	0	0	0	0	0	0	0	0
5–29.99%	^a^	^a^	0	0	^a^	^a^	0	0	^a^	^a^
30–49.99%	^a^	^a^	^a^	^a^	0	0	^a^	^a^	0	0
≥50%	241	62.4	51	13.9	28	11.8	16	7.8	^a^	^a^
Bangladeshi										
0–4.99%	0	0	0	0	0	0	^a^	^a^	0	0
5–29.99%	^a^	^a^	^a^	^a^	0	0	^a^	^a^	^a^	^a^
30–49.99%	^a^	^a^	^a^	^a^	0	0	0	0	^a^	^a^
≥50%	77	74.0	^a^	^a^	^a^	^a^	^a^	^a^	^a^	^a^
Black Caribbean										
0–4.99%	0	0	0	0	0	0	0	0	^a^	^a^
5–29.99%	^a^	^a^	^a^	^a^	0	0	^a^	^a^	^a^	^a^
30–49.99%	^a^	^a^	^a^	^a^	^a^	^a^	^a^	^a^	^a^	^a^
≥50%	109	39.5	57	34.3	25	14.7	12	8.4	^a^	^a^
Black African										
0–4.99%	^a^	^a^	^a^	^a^	0	0	0	0	0	0
5–29.99%	^a^	^a^	^a^	^a^	^a^	^a^	^a^	^a^	0	0
30–49.99%	^a^	^a^	0	0	^a^	^a^	^a^	^a^	0	0
≥50%	86	50.8	26	18.9	17	15.9	13	11.8	^a^	^a^

The use of these data does not imply the endorsement of the data owner or the UK Data Service at the UK Data Archive in relation to the interpretation or analysis of the data. This work uses research datasets which may not exactly reproduce National Statistics aggregates

^a^Sample size smaller than 10—estimates can not be displayed due to confidentiality concerns

## Appendix 5

See Table 9.

**Table 9:** Description of area-level deprivation by level of own ethnic density in the Early Childhood Longitudinal Study-Birth Cohort, US

Ethnic groups	Multiple Deprivation Index quintiles									
	Quintile 1 (least deprived)		Quintile 2		Quintile 3		Quintile 4		Quintile 5 (most deprived)	
	*n*	Weighted%	*n*	Weighted%	*n*	Weighted%	*n*	Weighted%	*n*	Weighted%
Non-Hispanic Black or African American										
0–4.99%	*	29.6	*	29.2	*	25.1	*	13.5	*	2.7
5–29.99%	50	10.6	50	22.3	50	25.1	100	24.5	50	17.4
30–49.99%	*	0.5	*	0.8	50	19.0	100	46.5	50	33.1
≥50%	*	0	*	1.0	*	2.5	50	21.0	200	75.5
Hispanic										
0–4.99%	50	44.9	*	24.1	*	11.3	*	13.4	*	6.2
5–29.99%	50	12.2	100	27.7	100	29.8	50	15.6	50	14.8
30–49.99%	*	0	*	1.3	50	18.9	100	43.0	50	36.8
≥50%	*	0	*	0.4	*	1.8	50	18.2	250	79.6
Asian										
0–4.99%	100	26.6	50	25.2	50	19.4	50	16.9	*	11.9
5–29.99%	100	25.0	100	25.5	50	16.2	50	15.6	50	17.7
30–49.99%	*	10.7	*	18.0	50	34.6	*	28.9	*	7.8
≥50%	*	0	*	23.4	*	41.2	*	17.1	*	18.3
American Indian and Alaska Native										
0–4.99%	*	3.8	*	22.3	50	22.9	50	37.2	*	13.8
5–29.99%	*	0	*	9.9	*	16.9	*	31.6	*	41.5
30–49.99%	*	0	*	0.0	*	0	*	81.6	*	18.4
≥50%	*	0	*	0.0	*	0	*	5.6	50	94.4

Unweighted sample sizes are rounded to the nearest 50

* <25
